# Incidence of Zika Virus Infection from a Dengue Epidemiological Study of Children in Ratchaburi Province, Thailand

**DOI:** 10.3390/v13091802

**Published:** 2021-09-10

**Authors:** Pimolpachr Sriburin, Pichamon Sittikul, Nathamon Kosoltanapiwat, Salin Sirinam, Watcharee Arunsodsai, Chukiat Sirivichayakul, Kriengsak Limkittikul, Supawat Chatchen

**Affiliations:** 1 Department of Tropical Pediatrics, Faculty of Tropical Medicine, Mahidol University, Bangkok 10400, Thailand; pimolpachr.srb@mahidol.ac.th (P.S.); pichamon.sit@mahidol.ac.th (P.S.); salin.sir@mahidol.ac.th (S.S.); watcharee.cho@mahidol.ac.th (W.A.); chukiat.sir@mahidol.ac.th (C.S.); kriengsak.lim@mahidol.ac.th (K.L.); 2 Department of Microbiology and Immunology, Faculty of Tropical Medicine, Mahidol University, Bangkok 10400, Thailand; nathamon.kos@mahidol.ac.th

**Keywords:** Zika virus, incidence, ELISA, RT-PCR, symptomatic infection, Thailand

## Abstract

Zika virus (ZIKV) is the mosquito-transmitted virus that the WHO declared a Public Health Emergency of International Concern in 2016 due to the consequence of microcephaly from infected pregnancies. The incidence of Zika infection has been unclear in many countries because most infected people have nonspecific febrile illnesses. This study’s aim is to investigate the incidence of symptomatic Zika virus infections from the archived samples of a dengue cohort study of children in central Thailand from 2006 to 2009. We performed Zika NS1 immunoglobulin (Ig)G enzyme-linked immunosorbent assay (ELISA) screening to identify symptomatic Zika infections in paired acute/convalescent serum samples. Symptomatic Zika infections were confirmed by reverse transcription polymerase chain reactions (RT-PCR) of acute serum samples. The comparison of the Zika NS1 IgG ELISA results between acute and convalescent samples showed 290/955 (30.4%) seropositive cases. Zika RT-PCR results were positive in 28 febrile cases (15 females, 13 males). Zika RT-PCR showed that symptomatic Zika infection occurred in children aged 4–11 years in Ratchaburi province, Thailand (2007–2009, first case in April 2007), and the symptomatic Zika:dengue infection ratio was 28 Zika:394 dengue (1:14). Phylogenetic analysis showed that all Zika viruses were of Asian lineage. Zika NS1 IgG ELISA identified Zika-infected patients and showed a low Zika:dengue ratio.

## 1. Introduction

Zika virus (ZIKV) is a mosquito-borne, single-stranded RNA virus in the genus *Flavivirus*, family *Flaviviridae*. ZIKV was first isolated from a rhesus monkey in the Zika forest in 1947 [1]. The first case of human infection was reported in Nigeria in 1954 [2]. In 1966, it was isolated from *Aedes africanus* mosquitoes in Malaysia [3]. The first outbreak of ZIKV infection was recognized on the Yap Islands in the Federated States of Micronesia in 2007 [4]. The ZIKV pandemic in Brazil started in Bahia, a northeastern state, and rapidly spread throughout the Americas [5]. More than 3000 cases of microcephaly were reported in Brazil, and isolation of ZIKV was confirmed from the brain tissue of an infected infant who died in the neonatal period [6]. In February 2016, the World Health Organization declared ZIKV infection to be a Public Health Emergency of International Concern [7].

ZIKV is transmitted to humans mainly through bites from infected mosquitoes of the genus *Aedes* [8]. In humans, the incubation period from mosquito bite to symptom onset is 3–12 days. ZIKV infection is mainly asymptomatic in 80% of cases [9,10]. When symptoms occur, they are typically mild, self-limiting, and nonspecific, similar to other arbovirus infections (e.g., dengue virus and chikungunya virus). Commonly reported symptoms include rash, low-grade fever, arthralgia, myalgia, fatigue, headache, and conjunctivitis [11]. Clinical evaluation alone is unreliable for the diagnosis of ZIKV infections.

In Southeast Asia, a seroprevalence study in the 1950s also showed that a percentage of the population was immune to ZIKV; however, the interpretation of serological results is complicated due to *Flavivirus* cross-reactivity [12]. ZIKV has been detected in travelers returning from Malaysia [13] and Thailand [14,15]. An investigation of ZIKV infection in the Thai population was conducted in 2012 [16]. However, the first virus isolation in Thailand was obtained from the archived serum of an acute ZIKV-infected patient in 2006 [17]. Molecular epidemiological and genetic diversity studies of ZIKV from mosquitoes and patients have shown that ZIKV has circulated at a low but sustained level within Thailand since at least 2002 [18,19].

The incidence rate of ZIKV infection remains unclear in many places, including Thailand. Non-structural protein 1 (NS1) in the ZIKV immunoglobulin (Ig)G enzyme-linked immunosorbent assay (ELISA) has shown potential as a serological test for the diagnosis of ZIKV infection [20,21]. We identified symptomatic ZIKV infections from the archived samples of a dengue cohort study in children of central Thailand from 2006 to 2009 [22]. Samples of the acute and convalescent sera (approximately 14 days apart) were collected from all febrile children in this cohort.

This study’s aim is to investigate the incidence of symptomatic ZIKV infections from the archived samples of a dengue cohort study of children in central Thailand from 2006 to 2009.

## 2. Materials and Methods

### 2.1. Study Site and Serum Samples

**Figure 1:**
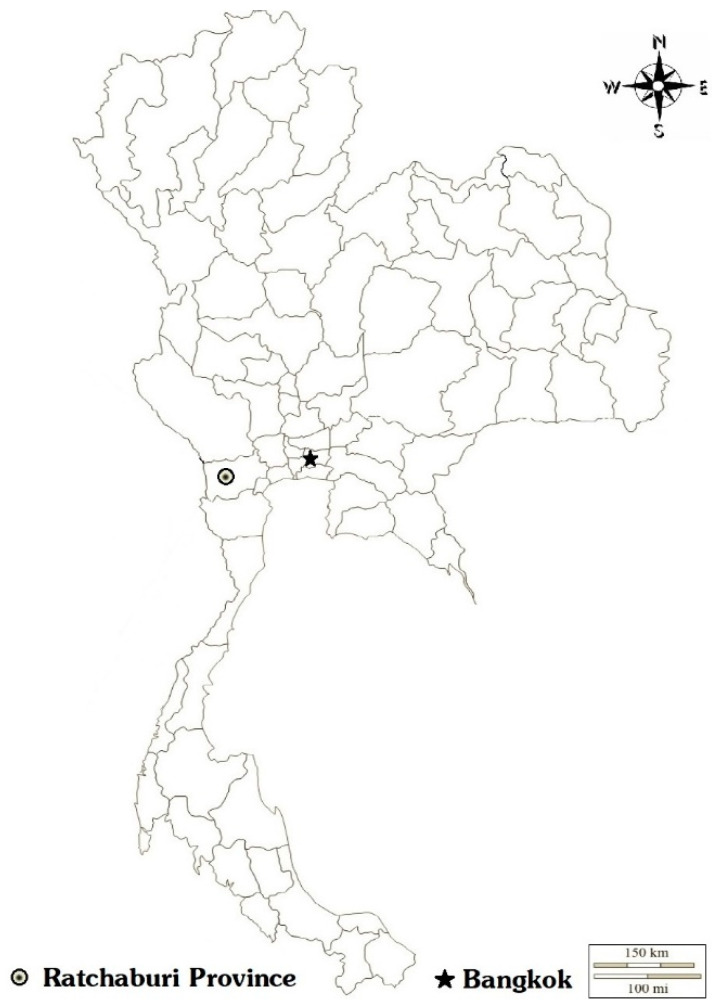
Participating Schools in Ratchaburi province, Thailand, 2006–2009. The circle indicates the location of schools, and the star indicates the location of the Faculty of Tropical Medicine, Mahidol University (Bangkok).

**Figure 2:**
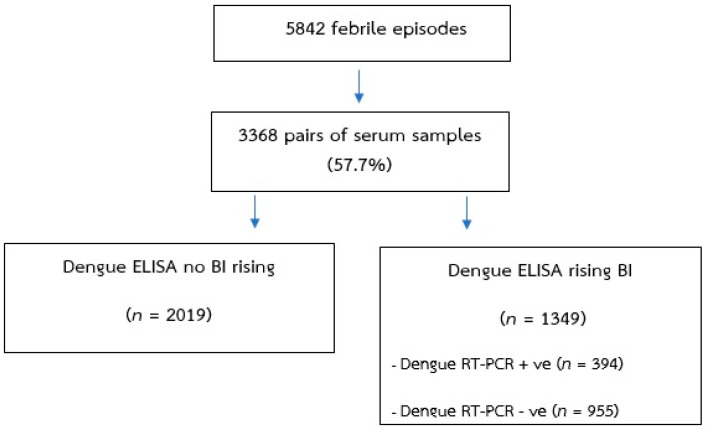
Schematic diagram of the study design and procedures. Specimens collected from children with acute febrile illness. All paired serum samples were used to perform dengue immunoglobulin (Ig)M/IgG enzyme-linked immunosorbent assay (ELISA) tests. The ELISA optical density results were transformed to binding index units.

In this study, archived serum samples (stored at −80 °C) from a cohort epidemiological study of dengue infection in school-aged children in Ratchaburi province, Thailand, from 2006 to 2009, were used (Figure 1). The epidemiology of dengue was investigated by active surveillance for acute febrile illness. In children with acute febrile illness, acute and convalescent serum samples were collected and tested for dengue, as described previously [22]. It was found that dengue accounted for 394 of the 5842 (6.74%) febrile episodes over the 4 years. As dengue–ZIKV cross-reactivity is commonly detected by ELISA, we selected acute and convalescent serum samples that showed an increase in serum dengue antibodies by ELISA but were also negative for dengue by reverse transcription polymerase chain reaction (RT-PCR), for this study. Additionally, paired acute and convalescent serum samples were tested for ZIKV NS1 IgG ELISA. The samples that showed an increase in ZIKV NS1 IgG ELISA optical density (OD) were further tested for ZIKV RT-PCR (Figure 2).

All data were handled confidentially and anonymously. This study was reviewed and approved by the ethics committee of the Faculty of Tropical Medicine, Mahidol University (protocol TMEC 17-072).

### 2.2. ZIKV NS1 IgG ELISA

ZIKV NS1 IgG ELISA, using acute and convalescent samples, was performed to evaluate the immune status of ZIKV patients [21]. Briefly, the 96-well ELISA plates were filled with 60 µL/well of ZIKV NS1 protein (500 ng) in a 0.018 M carbonate buffer. After overnight incubation at 4 °C, the plates were washed six times with 300 µL/well of phosphate-buffered solution containing 1% Tween 20 (PBS-T) and blocked with 5% skim milk in PBS-T for 1 h at 37 °C. After washing, the plates were filled with diluted serum (1:100) with buffer (PBS with 5% skim milk and 1% Tween 20) and controls. Fifty microliters per well of diluted serum was added in duplicate, and the plates were incubated at 37 °C for 1.5 h. After washing again, the plates were filled with 50 µL/well of a goat-anti-human IgG antibody conjugated to horseradish peroxidase (KPL Inc., Gaithersburg, MD, USA), diluted at 1:5000 in PBS-T with 5% skim milk. After incubation at 37 °C for 90 min, the plates were washed six times, and then the substrate SureBlue^™^ TMP (KPL Inc., Gaithersburg, MD, USA) was added. After incubation at room temperature for 30 min, the reaction was terminated by addition of 50 µL of 0.2 M sulfuric acid to each well. The ODs at 450 nm of the wells were measured by an ELISA plate reader. A convalescent:acute sera ELISA OD ratio of ≥1.2 was considered to be seropositive. The analysis of the ELISA OD ratio was performed with SPSS version 18.0 (SPSS, Inc., Chicago, IL, USA). Data were analyzed by Mann–Whitney U tests to determine the difference between groups, and a *p*-value < 0.05 was considered as statistically significant.

### 2.3. RT-PCR

For the ZIKV RT-PCR, the paired acute serum samples of ELISA-positive convalescent serum samples were selected for RNA extraction using a QIAGEN Viral RNA Kit (Qiagen, Hilden, Germany) according to the manufacturer’s instruction. All of the RNA samples were subjected to RT-PCR with primers (Table 1) [4], using a SuperScript^®^ III One-Step RT-PCR System with a Platinum^®^ Taq DNA Polymerase kit (Invitrogen, Carlsbad, CA, USA, Bio-Rad). The reaction mixture contained 1× reaction mix, 0.4 µM of each forward primer (ZIKV 835F) and reverse primer (ZIKV 1162R), 0.8 µL of SuperScript^®^ III RT/Platinum^®^ Taq Mix, and 2 µL of the total RNA in a final volume of 20 µL. The thermal cycling conditions were set as a reverse transcription at 50 °C for 30 min, followed by an initial denaturation of cDNA at 94 °C for 5 min, 35 cycles of denaturation at 94 °C for 15 s, annealing at 55 °C for 30 s, and an extension at 72 °C for 1 min, followed by a final elongation step 72 °C for 7 min. The PCR products of 327 bp for ZIKV were resolved on a 1.5% agarose gel, stained with ethidium bromide, and visualized under a gel documentation system (GelDoc^TM^ XR+ imaging system, Molecular Imager^®^, Bio-Rad, Hercules, CA, USA).

### 2.4. ZIKV Sequencing and Phylogenetic Analysis

The high yield of PCR products of the expected size (327 bp) was selected and purified from 1.5% agarose gel by using a QIAquick gel extraction kit (QIAGEN, Hilden, Germany) following the kit’s protocol. The purified DNA fragments were sent for DNA sequencing service at Macrogen Co., Ltd. (Seoul, Korea) using both primers. The sequencing chromatograms were inspected and processed by using BioEdit (Bioedit Ltd, Manchester, UK, v7.2.5) [23]. Nucleotide sequences were used as BLAST queries to search the National Center for Biotechnology Information (NCBI) database and aligned with reference sequences by using the ClustalW component of BioEdit. Phylogenetic trees were constructed by using Molecular Evolutionary Genetics Analysis (MEGA, v10, Pennsylvania State University, State College, PA, USA) software [24]. The maximum likelihood method with the K2 + G model was applied according to a phylogenetic model analysis [24]. Bootstrap resampling analysis of 1000 replicates was performed. The ZIKV genome sequences obtained from the GenBank database, NCBI, were used as reference sequences for phylogenetic analysis. The ZIKV fragments obtained in this study were submitted to the GenBank database. The accession numbers are MZ318404-MZ318420.

## 3. Results

### 3.1. Serum Samples

The epidemiology of dengue in school-aged children of Ratchaburi province, Thailand, from 2006 to 2009, was investigated by active fever surveillance for acute febrile illness. Over the 4-year study period, active surveillance for acute febrile illness was performed in on an average of 2753 primary school students (person-years). From active fever surveillance in the dengue cohort, 5842 febrile illness episodes were captured, with a mean number of febrile episodes of 0.53 per child per year. There were 3368 out of 5842 (57.7%) (paired sera collected and tested for dengue virus IgM/IgG by capture ELISA). Serum samples of dengue cases and febrile cases who had non-reaction from dengue ELISA tests were excluded. Therefore, 1910 acute and convalescent serum samples (28.4% or 955 of 3368) from children who had febrile illness were tested for ZIKV NS1 IgG ELISA. Furthermore, 290 acute serum samples from those who showed a rise in ZIKV NS1 IgG ELISA OD (seropositive) were selected to test for ZIKV RT-PCR.

### 3.2. ZIKV NS1 IgG ELISA

Both acute and convalescent serum samples from febrile illness (1910 samples from 955 febrile episodes) were tested for ZIKV NS1 IgG ELISA. Comparison of the acute and convalescent samples showed that 290 of 955 (30.4%) cases were seropositive (the rising OD ratio ≥ 1.2). The background antibody was further analyzed to understand the nature of our specimens. In addition, the background antibody from acute serum can be different in various populations due to the immune response of previous *Flavivirus* infections. Among these, 205 of 290 (70.7%) acute serum samples showed a low or naïve background for ZIKV NS1 IgG ELISA (OD <2 times the control OD).

**Figure 3:**
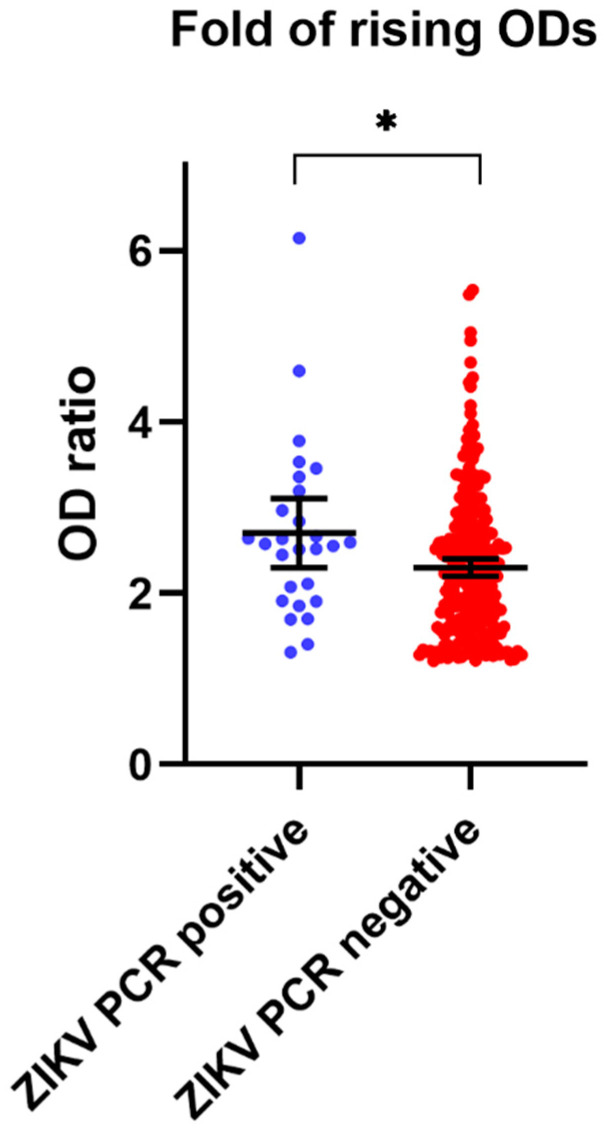
Rising OD folds between acute/convalescent serum samples. ZIKV NS1 IgG ELISA results of the analysis of 28 ZIKV (ZIKV RT-PCR confirmed) cases (blue) and 262 non-ZIKV infected persons (red). The OD ratio of the ZIKV group was statistically significantly different from that of the non-ZIKV group (* *p* = 0.022).

The ratios of the ELISA OD values of confirmed ZIKV and non-ZIKV groups are shown in Figure 3. The OD ratio of the ZIKV RT-PCR-positive group was statistically significant difference from that of the ZIKV RT-PCR-negative group (*p* = 0.022), with a mean OD ratio number of 3.5 (95% CI: 1.8–5.0) in the confirmed ZIKV group, and 2.6 (95% CI: 2.3–2.9) in the non-ZIKV group. Most of the ZIKV-positive cases showed OD ratios higher than 1.5; therefore, a potential cutoff criterion for the investigation of ZIKV infection in paired serum samples was set to ratios ≥1.5.

### 3.3. Incidence of ZIKV Infection

Among 290 febrile illness episodes with seropositive ELISA, ZIKV RT-PCR results were positive in 28 cases. Among the ZIKV-infected cases, 15 were females and 13 were males (mean age: 8 years; range: 5–12 years). The average body temperature at the first visit was 37.8 °C (maximum temp: 40.3 °C), and 12 of 28 reported body temperatures were >38 °C.

**Figure 4:**
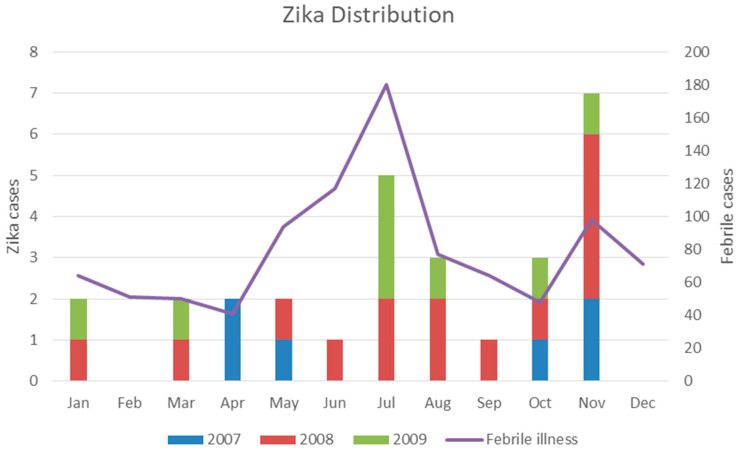
Distribution pattern of symptomatic ZIKV cases from school-aged children in Ratchaburi province, Thailand, from 2007 to 2009. ZIKV cases are shown in 2007 (blue), ZIKV cases in 2008 (red), and ZIKV cases in 2009 (green). The 955 febrile illness cases are shown as the purple line.

According to 394 cases of symptomatic dengue infection, the mean dengue incidence over the 4 years was 3.6/100 person-years. Considering 28 cases of symptomatic ZIKV infections in the same study area, the mean ZIKV incidence was approximately 2.5/1000 person-years, or the symptomatic ZIKV:dengue infection (Z:D) ratio in the population of Ratchaburi province, Thailand, in this period was approximately 1:14. These 28 ZIKV infections were found in five of seven schools participating in the cohort study. The first case of ZIKV infection was detected in April 2007. The numbers of ZIKV infections were 6, 14, and 8 in 2007, 2008, and 2009, respectively (Table 2, Figure 4). All ZIKV-infected patients had mild symptoms and self-recovered.

### 3.4. ZIKV Phylogenetic and Diversity

**Figure 5:**
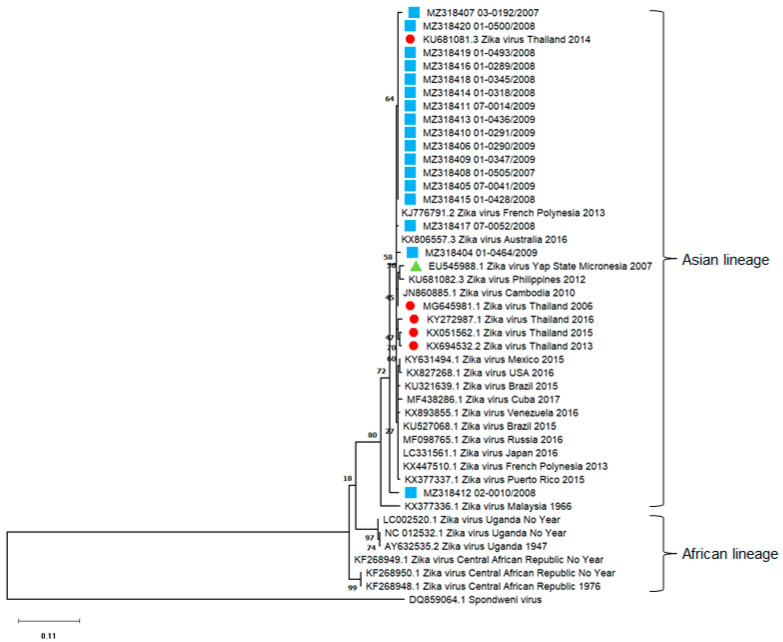
Phylogenetic analysis of ZIKV fragments (300 bp). The tree was constructed by using ZIKV sequences obtained from school-aged children in Ratchaburi province, Thailand, from 2007 to 2009 (blue square), ZIKV sequences from other studies in Thailand (red circle), ZIKV sequences (EU545988.1) from Yap Islands in Micronesia 2007 (green triangle). The maximum likelihood method with a bootstrap value of 1000 was applied. Sample names and nucleotide sequence accession numbers are marked with symbols. The Spondweni virus (DQ859064.1) sequence served as the out-group.

A phylogenetic tree was inferred by using ZIKV partial PrM and E genes sequence data from 17 of 28 (60.7%) ZIKV RT-PCR-positive samples. The diversity of ZIKVs was demonstrated throughout the study period (2007–2009). Our data suggested that the ZIKVs found in 2007 were different from those reported in the Yap Islands outbreak in 2007 (Figure 5).

## 4. Discussion

After acute ZIKV infection, IgM and IgG antibody responses occur, and the IgG level may be detectable for >1 year. Detection of a rising IgG level should be useful for the detection of recent ZIKV infections, both symptomatic and asymptomatic. Appropriate methods for detecting symptomatic ZIKV infection depend on the objectives, budgets, and feasibility. Although the plaque reduction neutralization test has been used as a gold standard serological test for >30 years, and is the most sensitive and specific method to detect *Flavivirus* infection, it still has some limitations, including its high cost, time-consuming nature, and the presence of cross-reactions between dengue and ZIKV infections. Several ZIKV immunoenzymatic assays have been developed with different formats. ELISA is an attractive alternative as an easy screening test for ZIKV infection with low cost [20,21,25,26].

The screening criteria for recent ZIKV infection using paired annual serum samples may be different because the rise in IgG level may decline and the baseline background antibody levels may be different. We evaluated the rise in ZIKV NS1 IgG ELISA OD values in 28 paired acute and convalescent serum samples from patients with symptomatic RT-PCR-proven ZIKV infection. To evaluate the potential cutoff screening criterion for ZIKV NS1 IgG ELISA, the rising OD ratios of the convalescent/acute samples were analyzed. Our results show that most of the symptomatic ZIKV subjects had a rise in ELISA ODs ≥1.5 fold. Only two subjects from the naïve group had a rise in ELISA ODs ranging from 1.2 to 1.49, due to gradually increasing antibody levels in the early phase after infection.

This indirect ELISA test can be used for screening in epidemiological studies or clinical settings for ZIKV infection [20,21,25]. For serological interpretation, the baseline and cross-reactivity are important factors. Therefore, the results from the pediatric population in this study may not reflect those of an adult population.

The ZIKV seroprevalence study conducted in Thailand during the 1950s indicated a possible circulation of ZIKV, but the results from serological testing alone may show cross-reactivity interference. The first evidence of a confirmed ZIKV case in Thailand was identified from the archived serum of an acute febrile patient in 2006 [17]. Since 2016, Thailand has established a centralized ZIKV surveillance system, but the previous incidence of ZIKV infection remains unclear [19]. The dengue cohort study of school-aged children in Ratchaburi province, central Thailand, from 2006 to 2009, obtained a mean dengue incidence of 3.6/100 person-years [22]. In the present study, the ZIKV incidence was very low (approximately 2.5/1000 person-years), as shown by the 1:14 symptomatic ZIKV:dengue infection ratio.

The first ZIKV case was identified in April 2007, and six ZIKV cases were detected from three schools in 2007. All ZIKVs showed different sequences, and the phylogenetic analysis showed genetic diversity during the 2007–2009 period, indicating that ZIKV had circulated at a low but sustained level within Ratchaburi province, Thailand, for a long time.

One limitation in this study is that we used ZIKV IgG ELISA to screen the selected serum samples, and then confirmed by RT-PCR. Although cross-reactivity is commonly detected by ELISA, it may not cover all candidate ZIKV infection especially in naïve groups. Moreover, since RT-PCR is specific for the diagnosis of ZIKV infection, it may not be very sensitive, and as such, the real incidence may be higher. In addition, the serum samples in this study were from subjects with acute febrile illness, assuming that all ZIKV infections in this study were symptomatic. Considering that the asymptomatic ZIKV infections may be at least one fold of symptomatic infections [27], the actual incidence of ZIKV infections in the study area and study period should be at least two times that reported here. In addition, dengue and ZIKV co-infection has not been investigated in this study.

## 5. Conclusions

This study shows that ZIKV had been endemic in Ratchaburi province, Thailand, at least since 2007. Viral transmission has been much lower for ZIKV than for dengue. The ZIKV cases in Thailand were sporadic, and our results show no evidence of a large ZIKV infection outbreak during the study period.

## Figures and Tables

**Table 1:** PCR primers for amplification of DNA fragments in the PrM and E genes of ZIKV.

Primer Name	Sequence (5’–3’)	Position	Product Size (bp)
ZIKV 835F	TTG GTC ATG ATA CTG CTG ATT GC	835–1162	327
ZIKV 1162R	CCA CTA ACG TTC TTT TGC AGA CAT		

**Table 2:** Characteristics of ZIKV cases.

Year	ZIKV Cases	Sex		Age (Years)			
		Male	Female	5–6	7–8	9–10	11–12
2007	6	3	3	1	5	0	0
2008	14	7	7	1	7	6	0
2009	8	3	5	0	2	2	4
Total	28	13	15	2	14	8	4
